# Clinical significance of proliferative potential of occult metastatic cells in bone marrow of patients with breast cancer

**DOI:** 10.1038/sj.bjc.6601121

**Published:** 2003-07-29

**Authors:** J-Y Pierga, C Bonneton, H Magdelénat, A Vincent-Salomon, C Nos, P Pouillart, J-P Thiery

**Affiliations:** 1 Medical Oncology Department, Institut Curie, 75248 Paris Cedex 05, France; 2 UMR 144 CNRS, Institut Curie, 75248 Paris Cedex 05, France; 3 Translational Research Laboratory, Institut Curie, 75248 Paris Cedex 05, France; 4 Pathology Department, Institut Curie, 75248 Paris Cedex 05, France; 5 Surgery Department, Institut Curie, 75248 Paris Cedex 05, France

**Keywords:** breast cancer, bone marrow, cytokeratin, culture

## Abstract

There is increasing statistical evidence that the presence of tumour cells in bone marrow detected by immunocytochemistry represents an important prognostic indicator in breast cancer, but their individual capacity to become clinical metastases is unknown. The aim of this study was to assess the proliferative capacity of these occult metastatic cells in the bone marrow of patients with various stages of breast cancer. We obtained bone marrow aspirates from 60 patients with breast cancer before treatment with chemotherapy: 17 stage II, 12 stage III and 31 stage IV. After bone marrow culture for 6–34 days (median: 17 days) under specific cell culture conditions, viable epithelial cells were detected by cytokeratin staining in 40 patients (66%). Expansion of tumour cells was poorly correlated with tumour cell detection on primary screening (*P*=0.06). There was a nonsignificant correlation between the number and the presence of expanded tumour cells and the UICC stage of the patients. On primary screening, tumour cell detection was positive in 56% of patients and was correlated with clinical UICC stage (*P*=0.01). However, with a median follow-up of 23 months, expansion of tumour cells from bone marrow was associated with decreased patient survival (*P*=0.04), whereas the survival difference according to detection of CK-positive cells on primary screening was not statistically significant. In conclusion, viable tumour cells can be detected in the bone marrow of breast cancer patients. Their proliferative potential could be predictive of outcome and deserves further investigation.

The clinical importance of occult tumour cells in the bone marrow of breast cancer patients has been demonstrated in several prospective studies, and represents an independent prognostic factor for distant relapse and overall survival (Harbeck *et al*, 1994; Diel *et al*, 1996; Mansi *et al*, 1999; Braun *et al*, 2000b; Gebauer *et al*, 2001; Gerber *et al*, 2001). Immunocytochemical (ICC) detection of epithelial cells in the bone marrow of breast cancer patients has been performed with a variety of antibodies, but the specificity and clinical relevance of the markers used to characterise epithelial cells remain controversial (Braun *et al*, 1998; Funke and Schraut, 1998). However, the simple detection of CK-positive cells does not provide any information about the proliferative potential of disseminated cancer cells. No marker is available to differentiate between cells that will die, those in a quiescent state and those with metastatic and proliferative potentials. Very little is known about the biological features of these cells. Currently available data suggest that CK-positive cells in bone marrow aspirates of cancer patients represent a selected, but nevertheless heterogeneous population of dormant (G0-phase) cancer cells (Braun and Pantel, 1999). Only a small fraction of disseminated tumour cells in bone marrow expressed a proliferative marker (Ki-67 or p120) in double-staining studies (Pantel *et al*, 1993). The dormant state of these cells may be one explanation for the relative resistance of micrometastatic tumour cells to chemotherapy (Braun *et al*, 2000a).

In a recent study, Solakoglu *et al* (2002) cultured bone marrow samples from 153 patients with various types of carcinoma (breast, prostate, colon and kidney). Viable epithelial cells were detected by cytokeratin staining in 81% of patients with no known distant metastases. Marked expansion of tumour cells was also correlated with decreased patient survival. Extensive cell culture experiments have also shown that cells disseminating into bone marrow have a time-limited proliferative potential (Pantel *et al*, 1995).

The present study is an attempt to investigate the clinical relevance of the *in vitro* proliferative potential of CK-positive tumour cells by culturing under specific tissue culture conditions and to compare the detection of these expended cells to ‘standard’ ICC detection in bone marrow using anticytokeratin antibodies.

## PATIENTS AND METHODS

### Patients

Sixty consecutive patients with primary or metastatic breast cancer were included in the study between 1999 and 2001 after giving their written informed consent. All patients were treated at the Institut Curie and follow-up data were obtained prospectively. The clinicopathological data and information concerning treatment modalities of the patients are summarised in Table 1

**Table 1:** Patient characteristics

			**Positive primary screening**			**Positive culture screening**		
***N*=60**	**No.**	**%**	**No.**	**%**	***P***	**No.**	**%**	***P***
Age (year), median, range	52	(36–74)	52 vs 52.7		0.74*	52 vs 52	0.98*	
*Menopausal status*								
Pre	32	54	18	56	0.94	22	69	0.71
Post	28	46	16	57		18	64	
								
*Clinical stage (UICC)*								
II	17	28	5	29.5	0.01	9	53	0.33
III	12	20	6	50		8	66.6	
IV	31	52	23	74.2		23	74.2	
								
*Hormonal status*^a^								
ER+	39	65	21	54	0.60	25	64	0.88
ER−	18	30	11	61		12	67	
Unknown	3	5						
PR+	19	32	13	68	0.55	15	79	0.24
PR−	24	40	12	50		15	62.5	
Unknown	17	28						
								
*Histology*^a^								
Ductal	51	83	30	59	0.42	33	65	0.43
Others	9	17	4	5		7	8	
								
*Tumour grade*^a^								
I	6	10	4	66	0.36	4	66	0.80
II	23	38	15	65		16	70	
III	20	33	9	45		12	60	
Unknown	11	18						

* Student's *t*-test.

a At initial diagnosis.

. Their median age was 52 years (range: 36–74 years). Histology showed 51 ductal, eight lobular, one apocrine and one undifferentiated carcinomas. Forty-one tumours were hormone receptor positive, 15 were negative and four were undetermined. UICC stages were 17 stage II, 12 stage III and 31 stage IV. TUICC classification of tumour sizes for nonmetastatic patients was three T1, 12 T2, two T3, four T4 not d, and six inflammatory breast cancers (T4d). Bone marrow sampling was performed in 14 patients before neoadjuvant chemotherapy, in 12 before adjuvant chemotherapy, based on four to six cycles of docetaxel–doxorubicin or epirubicin, cyclophosphamide ±5FU. Only three nonmetastatic patients did not receive adjuvant chemotherapy. The median follow-up was 23 months (range: 13–31 months) after sampling. Bone marrow aspirates (4–5 ml per sample) were obtained from both anterior iliac crests preoperatively in five patients or before starting chemotherapy (neoadjuvant or for metastatic disease) in 55 patients. One bone marrow aspirate was obtained under local anaesthesia from the posterior iliac crest (39 patients including all the stage IV patients) or by sternal tap (4–5 ml per sample) (16 patients).

### Bone marrow preparation

Three to five millilitres of bone marrow aspirate were collected and stored on EDTA (Vacutainer). Components of the bone marrow aspirate were processed under laminar flow. Each sample was diluted in Hanks (Gibco Brl UK) and separated by Ficoll/Hypaque density centrifugation (Sigma; density, 1.077 g ml^−1^) in Leucosep tubes (Polylabo) (830 **g**, 15 min, 20°C). The mononuclear cell (MNC) layer was harvested from each tube, combined, diluted in Hanks and centrifuged at 360 **g**, 5 min at 20°C in a 50 ml conical tube. The cells were resuspended in PBS solution +0.1% BSA (bovine serum albumin). Cell counts were performed on aliquots diluted in 3% acetic acid for red cell lysis. An aliquot of aspirate was tested for the presence of CK-positive tumour cells (primary screening). The remaining bone marrow cells were cultured and then rescreened for CK expression (culture screening).

### Primary screening

Mononuclear cells were resuspended at 1 × 10^6^ ml^−1^. One millilitre of the cell suspension (approximately 10^6^ cells) was cytocentrifuged onto polylysinated slides at 580 **g** twice for 3 min (Hettich Universal 16A cytocentrifuge) (Schwartz, 1991). The supernatant was carefully removed from each slide after the first cytocentrifugation and the slides were allowed to dry in air overnight. Slides were stored at room temperature before staining or were stored at −20°C and then at −80°C until staining.

### Cell culture and culture screening

Bone marrow cells were cultured as described by Pantel *et al* (1995) and Solakoglu *et al* (2002). MNCs (1 × 10^7^ to 3 × 10^7^) were initially plated in culture flasks coated with an extracellular matrix (Paesel & Lorei, Frankfurt, Germany). The culture medium contained RPMI 1640 supplemented with 10% FCS, 10 *μ*g ml^−1^ transferrin, 5 *μ*g ml^−1^ insulin, 2 mM glutamine, 10 ng ml^−1^ basic Fibroblastic Growth Factor (Boehringer Mannheim, Germany) and 10 ng ml^−1^ recombinant human epidermal growth factor (Boehringer Mannheim, Germany). The cells were cultured under 5% CO_2_ and reduced oxygen (4%). Before confluence, the adherent cells (including the epithelial tumour cells) were removed by trypsination. To determine the number of CK-positive cells, an aliquot containing 1 to 30 × 10^5^ cells was centrifuged onto glass slides and immunocytochemically stained (culture screening) using the method described above. In some cases, cells were transferred into new flasks. Two to three slides were stained with A45 B/B3 and one to two slides were used as controls with irrelevant IgG1. The number of *in vitro* expanded CK-positive cells was calculated: number of CK cells detected on slides after trypsination on culture screening according to the total number of MNCs per slide (from 10^4^ to 10^5^) divided by the total number of MNCs cultured at the time of sample collection. Results are expressed as the number of CK cells/10^6^ MNCs.

### Immunocytochemical staining

The pancytokeratin (CK) monoclonal antibody A45-B/B3 (Micromet, Germany and Chromavision, USA), which recognises several cytokeratin epitopes CK 8, CK 18 and CK 19, was applied for epithelial cell detection (Stigbrand *et al*, 1998). The immunostaining procedure was standardised by using a Cadenza (Shandon) automat. Before staining, cytospots were fixed with 4% paraformaldehyde for 5 min then dried for 15 min at room temperature. Endogenous alkaline phosphatase was then blocked with TBS solution with 2% AB serum (15 min) (Sanofi Diagnostics Pasteur, USA) and 2% levamisole. This solution was used to dilute primary and secondary antibodies. After blocking, the slides were incubated with the primary antibody A45 B/B3 for 40 min (2 *μ*g ml^−1^). Control slides were incubated under the same conditions with a mouse monoclonal anti-FITC IgG1 (one out of 1250) (Sigma Immuno Chemicals, USA). Slides were incubated for 20 min with secondary polyclonal rabbit anti-mouse antibody (Dako, USA). After each step, the slides were rinsed for 5 min in TBS 1 × solution. Immune complexes were revealed by the alkaline phosphatase-anti-alkaline phosphatase technique (Dako, USA) (one out of 50) for 25 min (Cordell *et al*, 1984). The chromogenic reaction was performed for 20 min with a colorimetric substrate of Fuchsin solution (2.5% in 2 N HCl) (New Fuchsin, Sigma) with 4% NaNO_2_, 8% *β*-naphthol (Sigma, USA) and 2% levamisole (Dako, USA). Cells were counterstained with Mayer's haematoxylin (1 min) (Sigma, USA) diluted to 1 : 3 in distilled water. The specimen was then rinsed under running water for 5 min and then in TBS. Slides were coverslipped using Faramount mounting medium (Dako, USA). Mononuclear cells (3 × 10^6^) were evaluated for each patient and for each bone marrow sample. Negative controls, stained with anti-FITC monoclonal mouse antibody, were performed on an equivalent number of cells (i.e. three slides, 3 × 10^6^ MNCs) for each patient.

Positive controls were obtained with bone marrow from normal donors undergoing orthopaedic surgery (Cochin Hospital), spiked with SKBR3 or MCF7 cell lines, 10–10^2^ for 10^6^ MNCs per cytospot. One positive control slide and one negative control slide were added to each series of 20 stained slides in the automated device.

### CK-positive cell detection by digital microscopy

The ACIS (ChromaVision Medical Systems, Inc.). is a computerised microscope, which includes an image processing system that has been optimised for the detection of rare carcinoma cells in specimens (Bauer *et al*, 2000). The application software supplied with the instrument starts by scanning a microscope slide at low magnification (× 10). The instrument then returns to objects originally identified by their stain for a second analysis at higher magnification (× 40 or × 60). In this case, more sophisticated image analysis of colour and morphometric characteristics is performed in order to exclude cellular debris, large clumps and cells with morphological features typical of normal haematological MNCs as opposed to CK-positive carcinoma cells. Cellular objects that meet colour- and morphometry-based criteria for probable tumour cells are collected and presented as montage images for review and classification by a pathologist or another investigator (JYP). Criteria for evaluation of immunostained cells in bone marrow were adapted from Borgen *et al* (1999) based on the results of the European ISHAGE Working Group for standardisation of tumour cell detection.

### Statistical methods

Patient characteristics were prospectively recorded on the Institut Curie medical files. Differences between treatment groups were analysed by *χ*^2^ tests for categorical variables and *t*-tests for continuous variables, and the Kruskal–Wallis test was used for nonparametric comparisons. Survival time and disease-free survival time were measured from the date of bone marrow aspiration until the date of death or last follow-up. Survival curves were determined using a Kaplan–Meier product-limit method (Kaplan and Meier, 1958). Statistical significance between groups was assessed using the log-rank test. Statistical analyses were performed by Statview software (SAS Institute Inc., 1998).

## RESULTS

### Expansion of bone marrow cells

**Figure 1:**
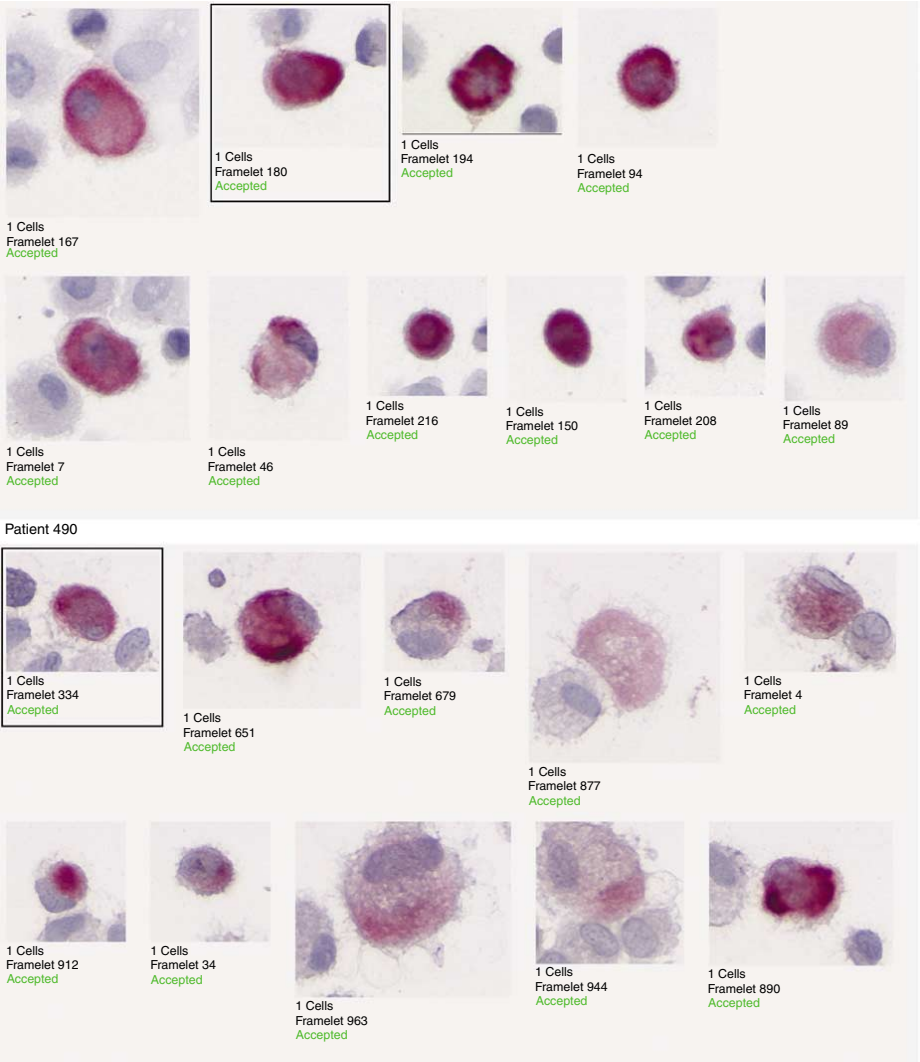
Example of automated detection and montage of CK-positive cells *in vitro* expanded bone marrow sample of two stage IV patients (patient's 344 and 490).

From August 1999 to March 2001, 60 bone marrow samples were successfully cultured. Cultures were stopped between 6 and 34 days (median: 17 days). An example of positive results is given in Figure 1

and Figure 2

**Figure 2:**
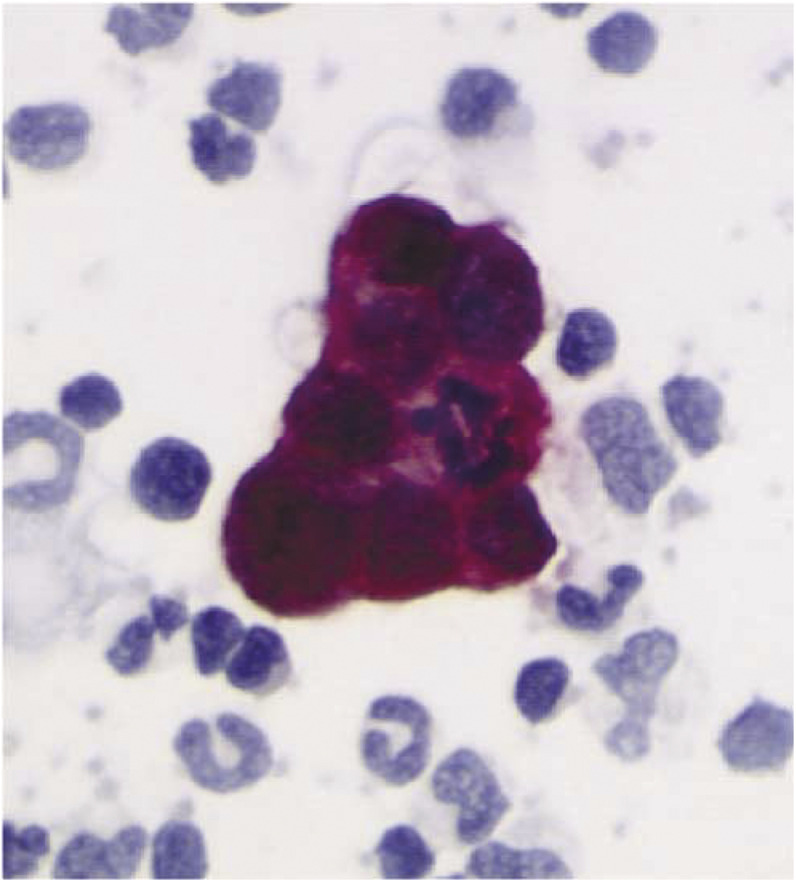
Cells in mitosis in a cluster detected in a stage IIB patient at primary screening.

. No correlation was observed between the number of CK-positive cells detected in the bone marrow sample (primary screening) and the number of CK-positive cells in the culture screening (*P*=0.19). Thirty-four (56%) patients presented a positive primary screening result, while 40 (66%) of these patients presented a positive culture screening result. CK-positive cell detection on primary screening was not strongly correlated with detection on culture screening (*P*=0.06). Fourteen cases (23%) initially negative on primary screening became positive on culture screening. Inversely, eight cases classified as positive on the initial screening, failed to produce any CK-positive cells (13%) on culture screening (Table 2

**Table 2:** Comparison of detection of CK-positive cells at initial screening and detection of CK-positive cells grown in culture of bone marrow, in 29 primary patients (stage I–III), in 31 metastatic patients (stage IV) and in the whole population (*N*=60)

	**CK-positive cells grown in culture**	**Negative primary screening**	**Positive primary screening**	
Primary	Negative	8	4	12 (41%)
(*N*=29)	Positive	10	7	17 (59%)
		18 (62%)	11 (38%)	*P*=0.66
				
Metastatic	Negative	4	4	8 (26%)
(N=31)	Positive	4	19	23 (74%)
		8 (26%)	23 (74%)	*P*=0.08
				
All patients	Negative	12	8	20 (33.3%)
(*N*=60)	Positive	14	26	40 (66.6%)
		26 (43.3)	34 (56.6)	*P*=0.065

).

### Correlation with tumour stage

The correlation between primary screening and tumour stage was statistically significant. Among the 34 patients with detectable CK-positive cells on primary screening, five of 17 (29%) had stage II tumours, six of 12 (50%) had stage III tumours and 23 of 31 (74%) had stage IV tumours, according to the UICC classification (International Union Against Cancer) (*χ*^2^ test, *P*=0.01). As shown in Table 3

**Table 3:** Correlation between UICC stage of the patients and number of CK-positive cells detected on 3 × 10^6^ MNCs on initial screening. Lack of correlation between UICC stage of the patients and extent of *in vitro* expansion of CK-positive cells

	**CK-positive primary screening**		**CK-positive culture screening**	
**Stage**	**Number of cells/3 × 10^6^**	**±s.d.**	**Number of cells/10^6^**	**±s.d.**
II (*n*=17)	1.5	3.9	10.2	24.1
III (*n*=12)	2	5.7	11.2	17.6
IV (*n*=31)	105.8	247.7	31.2	89.1
Total (*n*=60)	55.5	184.3	21.3	66.0
Kruskal–Wallis	*P*=0.0013		*P*=0.21	

, the number of CK-positive cells on initial screening was correlated with UICC stage (*P*=0.0013). No significant correlation was observed between the results of culture screening and tumour stage (*P*=0.21) (Table 3). Tumour cell expansion was not correlated with hormonal receptor status (*P*=0.88) or histology (ductal *vs* lobular) (*P*=0.43) or tumour grade (*P*=0.80) (Table 1). Interestingly, not all cell cultures of the 31 patients with clinically overt metastases harboured CK-positive cells: eight cases were negative for culture (26%) (Table 2). Cultures were also negative in four cases of clinical metastatic disease with positive initial screening (see Table 2).

### Correlation with clinical outcome

**Figure 3:**
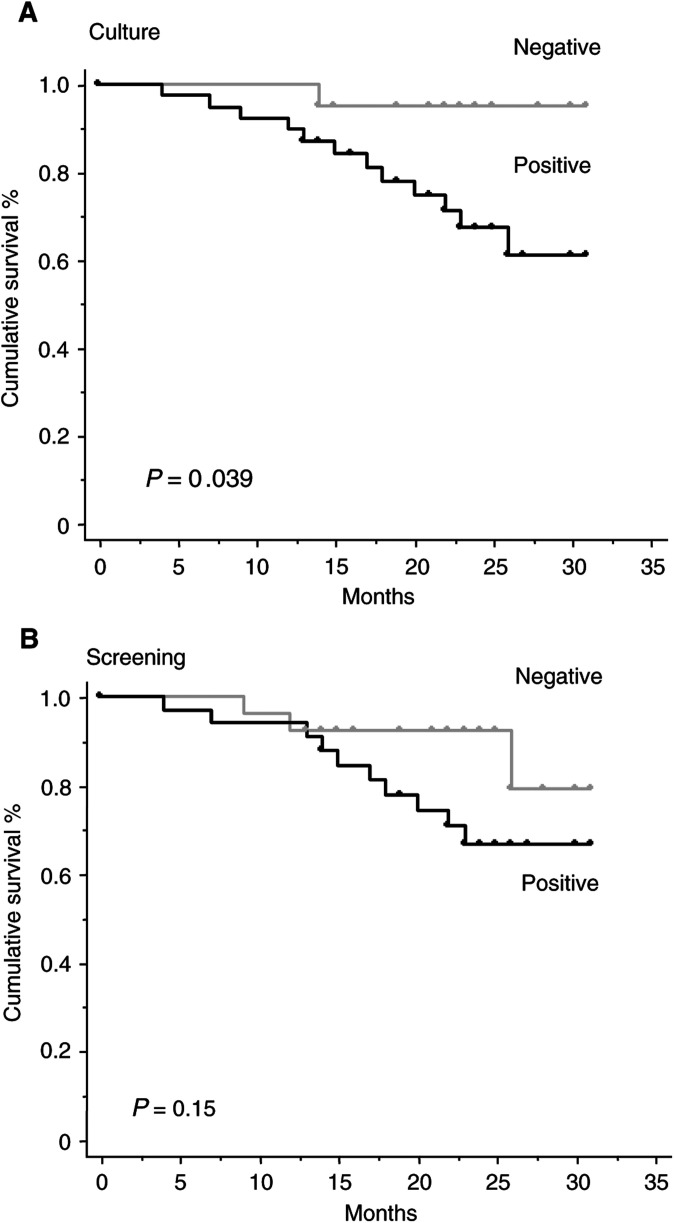
(**A**) Overall survival according to detection of CK-positive cells grown in culture of bone marrow (60 patients). (**B**) Overall survival according to the detection of CK-positive cells at initial screening of bone marrow sample (60 patients).

The median follow-up was 23 months. Thirteen deaths have occurred, all in metastatic patients. The median survival has not been reached in view of this short median follow-up. Statistical analysis (log-rank test) showed that primary detection of CK-positive cells in 34 patients was not significantly correlated with an increased risk of cancer-related death (*P*=0.15) (Figure 3B

). Forty patients had positive culture screening and 12 of them have died. Two of these patients had a negative primary screening. The difference in overall survival between the CK-positive group and the CK-negative group was statistically significant on Kaplan–Meier analysis (*P*=0.04) (Figure 3A).

## DISCUSSION

The present study investigates the clinical relevance of the extent of *in vitro* expansion of CK-positive cells present in bone marrow aspirate, under adapted conditions published by Pantel *et al* (1995) and Solakoglu *et al* (2002). Compared to Solakoglu's study, our study included more metastatic patients and a smaller total number of patients. However, our series is homogeneous in terms of the type of cancer, as all patients presented breast cancer, in contrast with Solakoglu's study which included a very heterogeneous population including prostate, renal and breast cancers. The detection of isolated tumour cells in the bone marrow of patients with metastatic breast cancer has also been demonstrated to be a factor of poor prognosis (Janni *et al*, 2000).

Cultured bone marrow cells may nonspecifically express CK detected by anticytokeratin antibodies. Pantel *et al* (1995) using the same antibody as that used in our study excluded this possibility by using a negative control and confirmation of tumour status of stained cells by FISH. Nonspecific immunostaining was eliminated in our series by staining the same number of control slides under the same conditions with a nonrelevant primary antibody (anti-FITC) for each case.

The number of *in vitro* expanded CK-positive cells was not correlated with the number of CK-positive cells on bone marrow aspirates. Pantel *et al* (1995) demonstrated the variable growth kinetics of these tumours. The number of CK-positive cells on culture screening may therefore not be related to the various bone marrow loads observed among individual patients, but could reflect the *in vitro* proliferative potential of these cells. The concentration of tumour cells in culture can then increase as a result of proliferation of tumour cells in culture of nonadherent bone marrow cells. A significant number of bone marrow samples (14 patients, 23%), negative on primary screening, became positive after cell culture. This could be explained by the number of bone marrow cells plated in culture flasks (10 to 30 × 10^6^) that is higher than the number of bone marrow cells examined on primary screening (3 × 10^6^). These cultures were grown under almost limiting dilution conditions, that is, fewer than 10 tumour cells were plated per flask, indicating that micrometastatic cells can inherit a strong growth potential. Culture techniques could increase the sensitivity of detection of occult tumour cells in human bone marrow about 100-fold (Joshi *et al*, 1990). In a previous study published by Ross *et al* (1993), ICC detection of tumour involvement in bone marrow and peripheral blood stem cell collections (PBSC) was significant with *in vitro* clonogenic growth (*P*<0.0001). The incidence and viability of tumour cell involvement in PBSC and bone marrow were studied in 48 patients with locally advanced or metastatic breast cancer enrolled on high-dose chemotherapy programmes. In culture experiments, clonogenic tumour colonies grew in 21 out of 26 immunocytochemically positive specimens. No tumour colony growth was detected in 30 out of 32 immunochemically negative specimens. In contrast, in our study, several cases positive on primary screening were negative after culture expansion. This could indicate that the cells initially detected were unable to proliferate, but it could also be due to failure of trypsinisation, leaving the tumour cells adherent to the culture flask. Examination of the slides after staining may also not completely reflect the contents of the culture flask. In comparison with native tumour cells, cells shed from a tumour were less clonogenic, more apoptotic and less tumorigenic (Swartz *et al*, 1999). The degree of anchorage-independent growth of tumour cells can predict their biological behaviour and metastatic potential *in vivo* (Li *et al*, 1989; O'Sullivan *et al*, 1999).

We found a statistically significant relationship between the *in vitro* extent of expansion of micrometastatic tumour cells and patient outcome, essentially the survival of metastatic patients. We did not observe any difference in survival or disease-free survival because of the small number of nonmetastatic patients in this series and the short follow-up. All the patients who died during the follow-up were the metastatic patients. The cells that were expanded may be sourced from the secondary tumour rather than the primary. However, the extent of *in vitro* expansion of CK-positive cells was a better prognostic indicator than simple detection of these cells in bone marrow samples. Similar data have been obtained for the expression of HER2 and urokinase plasminogen activator receptor in bone marrow (Heiss *et al*., 1995; Allgayer *et al*, 1997; Braun *et al*, 2001). Bone marrow cells were cultured under specific conditions: cultures were performed under hypoxic conditions (4% O_2_ instead of 20% O_2_), as hypoxia stimulates carcinoma invasiveness by upregulating uPAR expression on the cell surface (Graham *et al*, 1999).

Analysis of cultured micrometastatic tumour cells could provide a greater number of CK-positive cells available for subsequent molecular analyses. Analysis of cultured CK-positive cells could contribute to the identification of the metastatic stem cells responsible for the formation of overt metastases. Cell lines have also been established from bone marrow micrometastases (Rye *et al*, 1996; Putz *et al*., 1999), but the culture process could lead to selection of those cell clones that adapt best to the culture conditions.

In all the large published series, not all patients with CK-positive cells in bone marrow develop clinical metastases. CK-positive cells appear to have a heterogeneous phenotype (Braun *et al*, 1999) and their genotype has also been shown to be heterogeneous (Klein *et al*, 2002). Klein *et al* reported a high genetic variability in minimal residual cancer, particularly in terms of chromosomal imbalances by using comparative genomic hybridisation. Two individual tumour cells from the same patient may not be identical, supporting the concept of genetic instability.

Solakoglu *et al* (2002) has suggested that larger prospective clinical trials be performed in order to confirm the clinical relevance of the *in vitro* extent of expansion of CK-positive cells. This type of trial would be very difficult to organise in view of the very specific culture conditions and the fairly long time required for detection of tumour cells in culture. However, a better understanding of the biology of micrometastatic cells is necessary to increase the precision of detection in bone marrow.
